# Designing color in metallic glass

**DOI:** 10.1038/s41598-019-40014-w

**Published:** 2019-03-01

**Authors:** Jong Hyun Na, Kyung Hee Han, Glenn R. Garrett, Maximilien E. Launey, Marios D. Demetriou, William L. Johnson

**Affiliations:** 1 Glassimetal Technology, Inc, Pasadena, CA 91107 USA; 2 Department of Applied Physics and Materials Science, California Institute of Technology, Pasadena, CA 91125 USA

## Abstract

“Designing” metallic glasses to exhibit properties beyond those offered within the narrow composition ranges where glass formation is possible poses a formidable scientific challenge. This challenge may be tackled by forming composite structures comprising a metallic glass matrix and homogeneously precipitated dendrites, known as “metallic glass matrix composites” (MGMCs). In principle, MGMCs can be designed to exploit the attractive performance characteristics of the metallic glass while alleviating its negative undesirable attributes. In this work we introduce a MGMC development concept for designing color in metallic glass. MGMCs consisting of a white-gold metallic glass matrix with finely dispersed yellow-gold microdendrites are explored. A series of gold MGMCs is developed displaying uniform and visually-unresolved yellow colors over a broad range of chromaticity, along with high overall hardness. This design concept paves the way for the development of a new generation of metal alloys that combine advanced engineering performance with attractive cosmetic attributes.

## Introduction

Metallic glasses offer a wide range of attractive properties, including unique mechanical performance^1^ that sometimes leads to exceptional damage tolerance^2^, excellent corrosion resistance^3^, and processing capabilities unparalleled in other metals^4–9^. But these qualities are confined over very narrow compositional ranges where formation of the bulk metallic glass is possible^10,11^. As such, it is often difficult to “design” metallic glass alloys that offer an optimized combination of desired properties, particularly properties that are mutually exclusive in a metallic glass such as strength and toughness^12^. One known strategy to “design” such metallic glasses is by precipitating crystalline dendrites into the metallic glass, thus forming a so called “metallic glass matrix composite” (MGMC)^13,14^. In this concept, the overall composite properties are controlled by features of the dendritic microstructure rather than by the nominal alloy composition. With this approach, combinations of desirable properties may be “designed” in the MGMC by “fine tuning” the dendrite morphology and volume fraction. Damage-tolerant MGMCs have been developed demonstrating unusual combinations of strength and toughness^14,15^. The bulk of the MGMC development effort to date was directed to optimizing mechanical properties for engineering applications. However, other non-structural properties, like optical properties desirable in cosmetic applications, are rapidly gaining importance in the field. Here, we introduce a MGMC development approach that takes advantage of the glass-forming ability of gold-based alloys and the broad chromaticity range of gold-based solid solutions to design gold MGMCs with controlled color and high hardness.

Gold is widely used in luxury products such as jewelry, watches, and ornamental articles. Pure gold metal is soft, ductile, and is easily scratched and worn away. As such, gold is most widely used in an alloyed form. Gold alloys have been developed over centuries to exhibit combinations of optical properties (color and appearance), strength, hardness, corrosion and wear resistance to meet the requirements and needs of this application. Commonly used gold alloys are classified by hallmarking criteria that characterizes the weight fraction of the gold content. For commercial luxury products, meeting a specified hallmark is a basic requirement. Commercial gold alloys are further distinguished by their optical properties, more specifically their color. Gold alloys are classified broadly as “yellow gold”, “white gold”, “rose gold”, “green gold”, etc. The alloy color is controlled by combining various alloying elements with pure gold to form a gold alloy.

Bulk metallic glasses based on precious metals with hallmarked concentrations for potential use in commercial jewelry and luxury products have been reported over the last decade^16–19^. The development of these precious-metal glasses is motivated by a desire to combine the inherent appeal of the precious metal with the unique engineering properties (e.g. hardness, wear and corrosion resistance) and processability of a metallic glass. Metallic glass formation in gold-based alloy systems has been limited to a relatively narrow range of alloy compositions containing Si as well as other noble or near-noble metals such as Cu, Ag, and Pd (refs^17,20,21^). These Au-based monolithic metallic glasses exhibit critical rod diameters typically up to 5 mm, and an essentially “pale” white-gold color^22^. The white color is likely the result of the “bleaching” effect of Si in gold alloys. These glasses are also observed to tarnish and change surface color following exposure to ambient air^23^.

In this work we introduce a concept of designing color in gold-based metallic glasses by precipitating finely dispersed gold-rich microdendrites of chosen chromaticity in a white-gold metallic glass matrix to create a MGMC microstructure. We demonstrate that by controlling the color, volume fraction, size, and overall morphology of the dendrites, gold MGMCs with a visually uniform color of choice may be produced having significantly improved hardness compared to traditional gold alloys.

MGMCs are generally produced by quenching an initially molten glass former that first undergoes partial crystallization forming a semisolid, followed by vitrification of the remaining glass-forming liquid. The final microstructure consists of isolated dendritic crystals embedded in a continuous metallic glass matrix. These dendritic composites were first reported in (Zr,Ti)-Be glass forming systems^13^. The isolated dendritic inclusions are essentially single crystals having the lattice structure of the primary solid solution. The volume fraction of the dendritic phase can range from as low as 10% to as high as 90%, while characteristic sizes of the microstructural features (dendrite trunk and arm diameter, interdendritic spacing, etc.) can range from tens to hundreds of micrometers. While the microstructural feature size appears to be closely dependent on the cooling rate applied to quench the semisolid, the volume fraction of dendrites is controlled almost exclusively by the composition of the overall alloy. Calorimetry and composition analysis reveal that the compositions and phase fractions of the primary phase and the metallic glass matrix are consistent with thermodynamic phase equilibrium^23^. Specifically, the compositions and molar fractions of the dendritic and glassy phases are found to be consistent with a “lever rule” construction between a bcc primary phase and the eutectic liquid. The large lattice mismatch between the primary solid solution and the competing equilibrium intermetallics is believed to be behind the ability of this system to form MGMCs. Such mismatch prevents heterogeneous nucleation of the equilibrium intermetallics over the primary phase dendrites. This lattice mismatch is thought to arise primarily from the large atomic size difference between Be and (Zr,Ti). Aside from the (Zr,Ti)-Be eutectic system, the only other alloy system reported to form “equilibrium” MGMCs is the La-(Cu,Ni)-Al (ref.^24^), which also likely arises from the large atomic size difference between La and (Cu,Ni).

In this work it was anticipated that the lattice mismatch between the equilibrium crystal phases in the (Au,Ag)-Si system may also be large enough to enable formation of MGMCs. Unlike (Zr,Ti)-Be and La-(Cu,Ni), however, where such mismatch arises from a large atomic size difference, the mismatch in the (Au,Ag)-Si system is thought to arise from vastly different crystal structures of (Au,Ag) and pure Si. The primary (Au,Ag) phase is face-centered cubic with 12 nearest neighbors, while pure Si is a tetrahedrally-bonded diamond cubic structure having just 4 nearest neighbors. A preexisting face-centered-cubic (Au,Ag) dendrite would likely be an unfavorable template for the heterogeneous nucleation of diamond cubic Si. Such lattice mismatch would hence encourage vitrification of the liquid matrix leading to MGMC formation. It was also thought that certain elemental additions known to promote glass formation in the (Au,Ag)-Si system, such as Cu and Pd (ref.^17^), might further enhance the glass forming ability of the matrix provided that their concentrations in primary (Au,Ag) remain below their respective solubility limits.

Microstructural optimization in (Au,Ag)-Si MGMCs was implemented here to design an overall composite color. The apparent uniformity or non-uniformity of the overall color in a composite is controlled by the size scales characterizing the microstructure, such as the average dendrite trunk diameter, arm diameter, arm spacing, interdendritic spacing, etc. The smallest length scale resolvable by the naked human eye is typically on the order of the width of a fine hair, i.e. about 20–30 μm. Hence, microstructural features smaller than 20–30 μm should not be visually resolvable and the overall color of the composite will appear uniform and homogeneous. When this is achieved, the simple rule of mixtures (linear interpolation) can be used to approximate the apparent uniform color of the two-phase MGMC. For a visually-unresolved microstructure, the average CIELAB coordinates of the overall MGMC will be the volume-weighted average of those of the dendrite and matrix phases.

Using this concept, Au-based MGMCs with controlled color were designed with an ultra-fine visually-unresolved microstructure comprised of dendrites of primary-Au having the fcc structure of pure gold dispersed in a metallic glass matrix. The alloying additions to the primary-Au are chosen to produce Au-rich solid solutions having color varying from yellow, to red, rose, or green, etc., depending on the concentration of these alloy additions. As such, the dendrites may exhibit “yellow gold”, “rose gold” or other standard gold colors determined by control of the concentration of the dissolved solute metals in primary-Au. As the solubility of Si is very low in primary-Au, Si will strongly partition to the liquid matrix during the growth of primary-Au dendrites in the semisolid. Owing to this strong partitioning, the primary-Au dendrites will be essentially Si-free and display mechanical, optical, and color properties determined by the concentration of solute dissolved in primary-Au. While the metallic glass matrix may appear optically white, the primary-Au dendrites may be designed to have high chromaticity by choice of the overall alloy composition and knowledge of the partitioning effect of the other solute metals. As such, the primary-Au dendrites will control the overall color of the MGMC.

Primary-Au solid solutions with Ag and Cu as solutes are of interest here. Like Au, Ag has substantially zero Si solubility, and does not form stable silicides. On the other hand, Cu is known to promote bulk-glass formation in Au-Si (ref.^17^), and although Cu has a finite solubility of Si and forms stable silicides, its concentration can be limited such that the driving force for silicide formation remains small. A color-map of the ternary Au-Cu-Ag system that divides the alloy composition space into regions according to the optical appearance of the alloys is presented in Fig. S1 (Supplementary Information). The concentrations of Ag and Cu can be varied according to this ternary color map to design the color of the primary-Au phase, and by extension, the overall color of the MGMC. In principle, one can systematically vary the CIELAB a* and b* coordinates of the composite overall color by controlling the composition of primary Au-Cu-Ag. For example, it is apparent from Fig. S1, that increasing the Ag concentration in the overall alloy raises the CIELAB b* coordinate of the primary-Au phase, and enhances the overall yellow appearance of the composite. Using this approach, one may create MGMCs with desirable CIELAB coordinates falling in the category of “yellow” chromaticity. Similarly, reducing the overall Ag concentration in the overall alloy composition will raise the CIELAB a* coordinate and drop the b* coordinate of the primary-Au phase and overall composite. So doing increases the red chromaticity, and produces an overall composite color falling under the category of “rose gold”.

By exploring the quinary alloy system (Au,Cu,Ag)-Pd-Si around a eutectic composition and employing differential scanning calorimetry, x-ray diffraction, scanning electron microscopy, and secondary ion mass spectroscopy, we identified a compositional “tie line” that connects a quinary eutectic glass-forming alloy with a primary-Au alloy. Specifically, the quinary eutectic Au_50_Cu_25.5_Ag_3_Pd_3_Si_18.5_ and the ternary Au_65.2_Cu_22.4_Ag_12.4_ were determined to be in thermodynamic phase equilibrium. It follows that any alloy whose composition is a linear interpolation between Au_50_Cu_25.5_Ag_3_Pd_3_Si_18.5_ and Au_65.2_Cu_22.4_Ag_12.4_ would be a MGMC having a microstructure comprised of Au_65.2_Cu_22.4_Ag_12.4_ dendrites embedded in a continuous metallic glass matrix with composition Au_50_Cu_25.5_Ag_3_Pd_3_Si_18.5_. For such MGMCs, the respective molar fractions of the metallic glass and primary-Au phases are determined by applying the “lever rule” along the compositional “tie line”. Hence, the compositions of the MGMCs follow the composition formula:

1 $${{\rm{Au}}}_{{\rm{65.2}}-{\rm{15.2}}{x}}{{\rm{Cu}}}_{{\rm{22}}\mathrm{.4}+3{\rm{.1}}{x}}{{\rm{Ag}}}_{{\rm{12}}\mathrm{.4}-9{\rm{.4}}{x}}{{\rm{Pd}}}_{{\rm{3}}{x}}{{\rm{Si}}}_{{\rm{18.5}}{x}}$$

with *x* ranging between 0 and 1 representing the molar fraction of the metallic glass phase within the MGMC.

Rapidly quenched rod samples and plate coupons of various alloys that follow the tie-line composition formula of Eq. (1) were prepared (see Methods). The alloys include a primary-Au phase alloy with composition Au_65.2_Cu_22.4_Ag_12.4_, three MGMC alloys with compositions Au_60_Cu_23.5_Ag_9.1_Pd_1_Si_6.4_, Au_58_Cu_24_Ag_7.5_Pd_1.5_Si_9_, and Au_55.5_Cu_24.4_Ag_6.2_Pd_2_Si_11.9_, and a metallic glass phase alloy with composition Au_50_Cu_25.5_Ag_3_Pd_3_Si_18.5_. The molar fractions of the metallic glass phase in the alloys, *x*, are 0, 0.35, 0.5, 0.65, and 1.0, respectively. The critical rod diameters of the MGMCs are evaluated to be approximately 4 mm, while that of the metallic glass phase alloy is found to exceed 5 mm. The Au weight fraction in all alloys exceeds 85%, thus satisfying the 18-karat hallmark (though it may be somewhat higher than desired in commercial jewelry).

**Figure 1:**
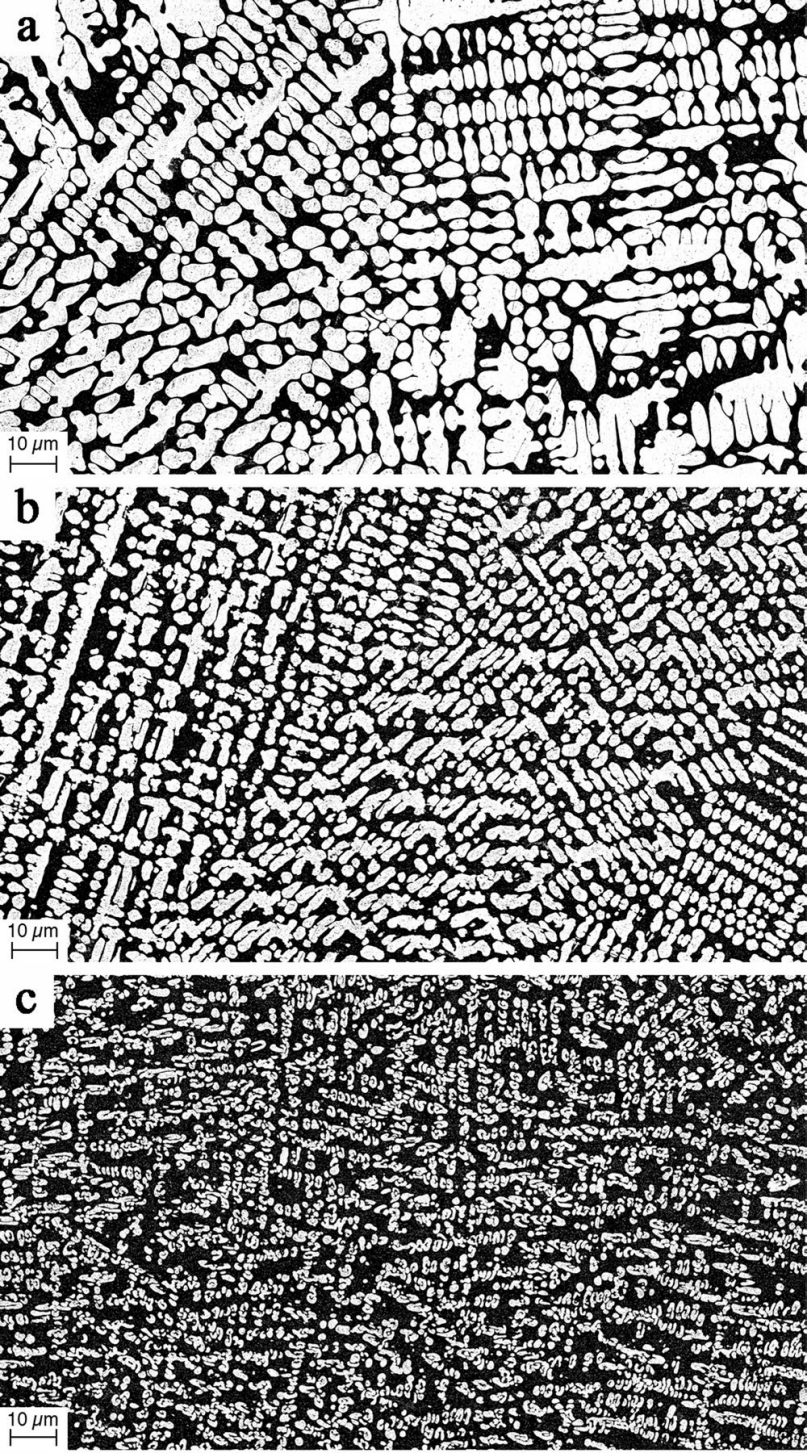
Scanning electron micrographs revealing the microstructure morphology of the MGMCs. (**a**) Au_60_Cu_23.5_Ag_9.1_Pd_1_Si_6.4_, (**b**) Au_58_Cu_24_Ag_7.5_Pd_1.5_Si_9_, and (**c**) Au_55.5_Cu_24.4_Ag_6.2_Pd_2_Si_11.9_. The dark-colored regions represent the metallic glass matrix phase while the light-colored represent the primary-Au particulate phase.

The microstructure morphology of the MGMCs Au_55.5_Cu_24.4_Ag_6.2_Pd_2_Si_11.9_, Au_58_Cu_24_Ag_7.5_Pd_1.5_Si_9_, and Au_60_Cu_23.5_Ag_9.1_Pd_1_Si_6.4_ is investigated by scanning electron microscopy performed on finely-polished cross sections of 2-mm diameter rods. Micrographs are presented in Fig. 1. The dark-colored regions are the metallic glass matrix phase while the light-colored is the primary-Au dendritic phase. No other phases are detectable in the micrographs. Using secondary ion mass spectroscopy, the compositions of the matrix and particulate phases in all MGMCs were verified (within expected error) to be Au_50_Cu_25.5_Ag_3_Pd_3_Si_18.5_ and Au_65.2_Cu_22.4_Ag_12.4_, in accordance with Eq. (1). The dendrites appear to be uniformly and homogeneously distributed through the matrix. The dendrite trunks appear to have developed radially along the direction of the temperature gradient established during the quench of the sample. Visually, the volume fraction of the metallic glass phase appears to increase roughly linearly with *x* (i.e. going from Au_60_Cu_23.5_Ag_9.1_Pd_1_Si_6.4_ to Au_58_Cu_24_Ag_7.5_Pd_1.5_Si_9_ to Au_55.5_Cu_24.4_Ag_6.2_Pd_2_Si_11.9_), in accord with the “tie line” construction assumed in Eq. (1). Lastly, the micrographs reveal fairly uniform and rather fine MGMC microstructures, displaying features (i.e. dendrite arm and trunk diameters, interdendritic spacing, etc.) characterized by an average size of less than ~5 μm. Specifically, the average dendrite trunk and dendrite arm diameters appear to be ~3–5 μm in Au_60_Cu_23.5_Ag_9_Pd_1.1_Si_6.4_, (Fig. 1a), ~2–4 μm in Au_58_Cu_24_Ag_7.5_Pd_1.5_Si_9_ (Fig. 1b), and ~1–3 μm in Au_55.5_Cu_24.4_Ag_6.2_Pd_2_Si_11.9_ (Fig. 1c) while the average interdendritic spacing appears to be ~1–3 μm in Au_60_Cu_23.5_Ag_9_Pd_1.1_Si_6.4_, (Fig. 1a), ~2–4 μm in Au_58_Cu_24_Ag_7.5_Pd_1.5_Si_9_ (Fig. 1b), and ~4–6 μm in Au_55.5_Cu_24.4_Ag_6.2_Pd_2_Si_11.9_ (Fig. 1c). It is noted that for each MGMC composition, the microstructural morphology remains roughly unchanged when changing the sample geometry or size (e.g. plates with thickness of up to 2 mm or rods with diameters of up to 4 mm), suggesting a minimal effect of cooling rate on the microstructure morphology.

**Figure 2:**
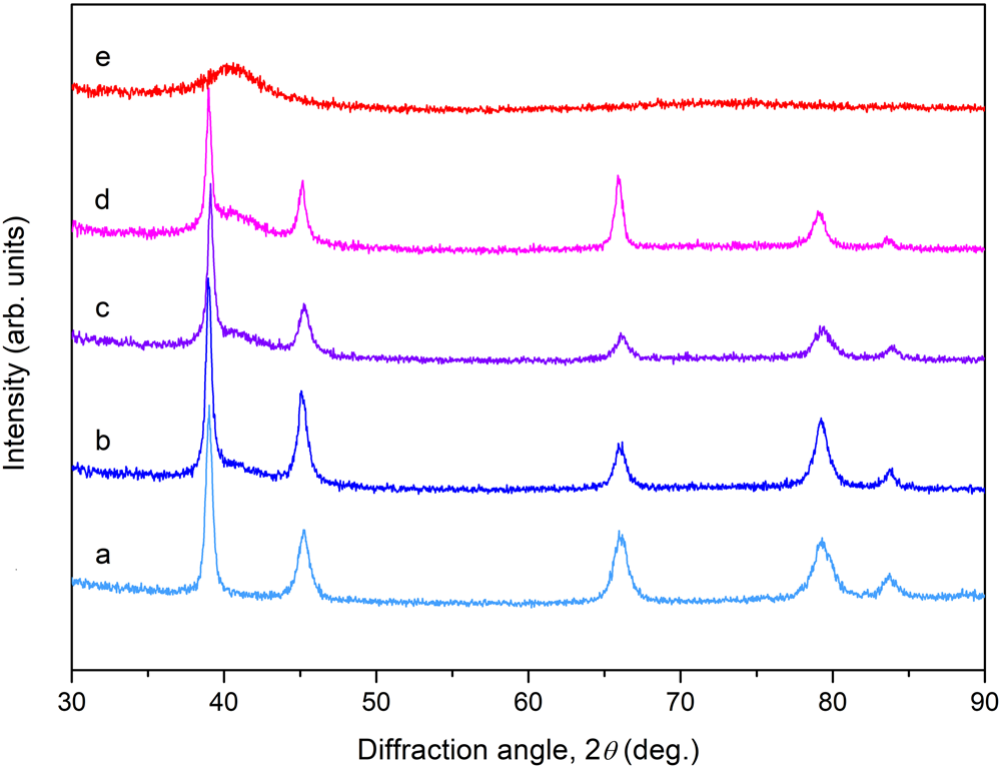
X-ray diffractograms revealing the atomic structure of the primary-Au, MGMC, and metallic glass alloys. (**a**) Au_65.2_Cu_22.4_Ag_12.4_, (**b**) Au_60_Cu_23.5_Ag_9.1_Pd_1_Si_6.4_, (**c**) Au_58_Cu_24_Ag_7.5_Pd_1.5_Si_9_, (**d**) Au_55.5_Cu_24.4_Ag_6.2_Pd_2_Si_11.9_, and (**e**) Au_50_Cu_25.5_Ag_3_Pd_3_Si_18.5_. The diffractograms verify the amorphous structure of the metallic glass phase alloy Au_50_Cu_25.5_Ag_3_Pd_3_Si_18.5_, the primary-Au structure of the particulate phase alloy Au_58_Cu_24_Ag_7.5_Pd_1.5_Si_9_, and composite structure of the MGMC alloys Au_60_Cu_23.5_Ag_9.1_Pd_1_Si_6.4_, Au_58_Cu_24_Ag_7.5_Pd_1.5_Si_9_, and Au_65.2_Cu_22.4_Ag_12.4_.

X-ray diffraction analysis was performed on finely-polished cross sections of 2-mm dimeter rods of the MGMCs Au_60_Cu_23.5_Ag_9.1_Pd_1_Si_6.4_, Au_58_Cu_24_Ag_7.5_Pd_1.5_Si_9_, and Au_55.5_Cu_24.4_Ag_6.2_Pd_2_Si_11.9_, the monolithic metallic glass Au_50_Cu_25.5_Ag_3_Pd_3_Si_18.5_, and the primary-Au Au_65.2_Cu_22.4_Ag_12.4_. X-ray diffractograms are presented in Fig. 2. The diffractogram of the metallic glass alloy Au_50_Cu_25.5_Ag_3_Pd_3_Si_18.5_ exhibits a diffuse halo pattern and no crystallographic peaks, consistent with a fully amorphous phase. The diffractogram of the primary-Au alloy Au_65.2_Cu_22.4_Ag_12.4_ exhibits crystallographic peaks consistent with a crystalline solid-solution having the face-centered cubic (fcc) structure of pure Au and no halo background, confirming the absence of any amorphous phase. The diffractograms of the MGMCs Au_60_Cu_23.5_Ag_9_Pd_1.1_Si_6.4_, Au_58_Cu_24_Ag_7.5_Pd_1.5_Si_9_, and Au_55.5_Cu_24.4_Ag_6.2_Pd_2_Si_11.9_ reveal a primary-Au fcc phase coexisting with the metallic glass phase. No peaks other than those consistent with the primary-Au fcc phase are evident, confirming the absence of any other crystalline (e.g. an intermetallic) phases. As *x* increases from 0.35 to 0.65 the intensity of the diffuse halo increases, suggesting that the molar fraction of the metallic glass phase increases at the expense of the primary-Au phase, consistent with the “tie line” construction given by Eq. (1).

**Figure 3:**
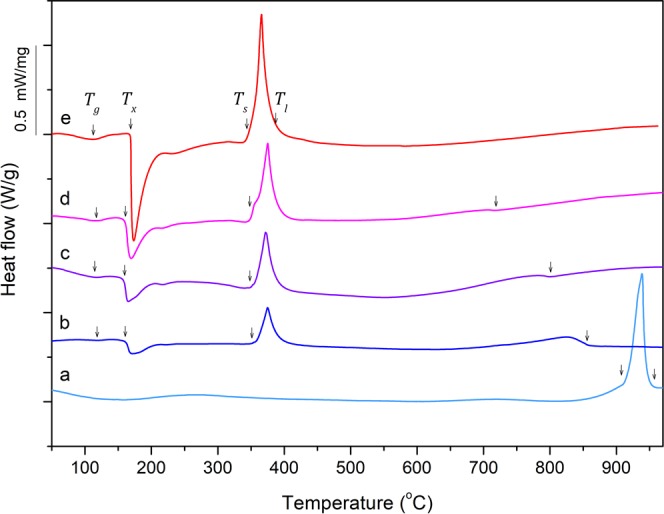
Differential scanning calorimetry of the primary-Au, MGMC, and metallic glass alloys. (**a**) Au_65.2_Cu_22.4_Ag_12.4_, (**b**) Au_60_Cu_23.5_Ag_9.1_Pd_1_Si_6.4_, (**c**) Au_58_Cu_24_Ag_7.5_Pd_1.5_Si_9_, (**d**) Au_55.5_Cu_24.4_Ag_6.2_Pd_2_Si_11.9_, and (**e**) Au_50_Cu_25.5_Ag_3_Pd_3_Si_18.5_. The glass transition temperature *T*_*g*_, crystallization temperature *T*_*x*_, solidus temperature *T*_*s*_, and liquidus temperature *T*_*l*_ for each alloy are indicated by arrows. *T*_*g*_, *T*_*x*_, and *T*_*s*_ are roughly constant between the monolithic metallic glass and MGMC alloys, but *T*_*l*_ rises considerably as the alloys transition from a pure metallic glass phase to a pure primary-Au phase.

Differential scanning calorimetry was performed on discs sectioned from 2-mm diameter rods of the MGMCs Au_60_Cu_23.5_Ag_9_Pd_1.1_Si_6.4_, Au_58_Cu_24_Ag_7.5_Pd_1.5_Si_9_, and Au_55.5_Cu_24.4_Ag_6.2_Pd_2_Si_11.9_, the monolithic metallic glass Au_50_Cu_25.5_Ag_3_Pd_3_Si_18.5_, and the primary-Au Au_65.2_Cu_22.4_Ag_12.4_. Calorimetry scans are presented in Fig. 3. The glass transition temperature *T*_*g*_, crystallization temperature *T*_*x*_, solidus temperature *T*_*s*_, and liquidus temperature *T*_*l*_ for each alloy are indicated by arrows in Fig. 3 (see Table S1, Supplementary Information). As seen in Fig. 3, *T*_*g*_ and *T*_*x*_ change negligibly between the monolithic metallic glass and the glassy matrix of the MGMCs, having values of about 115 °C and 160 °C, respectively. *T*_*s*_ of the metallic glass and MGMCs alloys is also shown to be roughly constant at 350 °C among these alloys, representing the eutectic temperature of this alloy system. On the other hand, *T*_*l*_ drops dramatically as the alloys transition from the pure primary-Au phase to the pure metallic glass phase (i.e. with *x* varying between 0 and 1). Specifically, *T*_*l*_ decreases from about 950 °C for the primary-Au Au_65.2_Cu_22.4_Ag_12.4_ to 375 °C for the metallic glass Au_50_Cu_25.5_Ag_3_Pd_3_Si_18.5_. Using the data for the receding liquidus temperature as a function of *x* along with the eutectic temperature of 350 °C, a pseudo-binary eutectic phase diagram is constructed along the “tie line” described in Eq. (1) to represent the phase equilibria between primary Au_65.2_Cu_22.4_Ag_12.4_ and eutectic Au_50_Cu_25.5_Ag_3_Pd_3_Si_18.5_ (see Fig. S2, Supplementary Information).

To verify that Eq. 1 indeed governs the compositions and respective molar fractions of the metallic glass and primary-Au phases in the MGMC’s, secondary ion mass spectroscopy is performed. The composition of the metallic glass phase in all MGMCs is identified to be Au 50.04 ± 0.18, Cu 25.30 ± 0.09, Ag 3.06 ± 0.08, Pd 3.06 ± 0.29, Si 18.53 ± 0.15 (at.%), while that of primary-Au is Au 65.21 ± 0.18, Cu 22.39 ± 0.63, Ag 12.39 ± 0.41, Pd 0.01 ± 0.02, Si 0.00 ± 0.00 (at.%). A round-off analysis suggests that the compositions of the metallic glass and primary-Au phases are Au_50_Cu_25.5_Ag_3_Pd_3_Si_18.5_ and Au_65.2_Cu_22.4_Ag_12.4_, respectively, consistent with the tie-line construction given by Eq. (1) and illustrated in the phase diagram of Fig. S2 (Supplementary Information). Through conservation of species, Eq. (1) provides a self-consistent relationship between the compositions and respective molar fractions of the phases in thermodynamic equilibrium, Au_50_Cu_25.5_Ag_3_Pd_3_Si_18.5_ and Au_65.2_Cu_22.4_Ag_12.4_. Hence, validating the compositions of the two phases also confirms that parameter *x* is an accurate representation of the respective molar fractions. The composition analysis performed here therefore verifies that the molar fractions of the metallic glass phase in MGMC’s Au_60_Cu_23.5_Ag_9_Pd_1.1_Si_6.4_, Au_58_Cu_24_Ag_7.5_Pd_1.5_Si_9_, and Au_55.5_Cu_24.4_Ag_6.2_Pd_2_Si_11.9_ are 0.35, 0.5, and 0.65, respectively.

**Figure 4:**
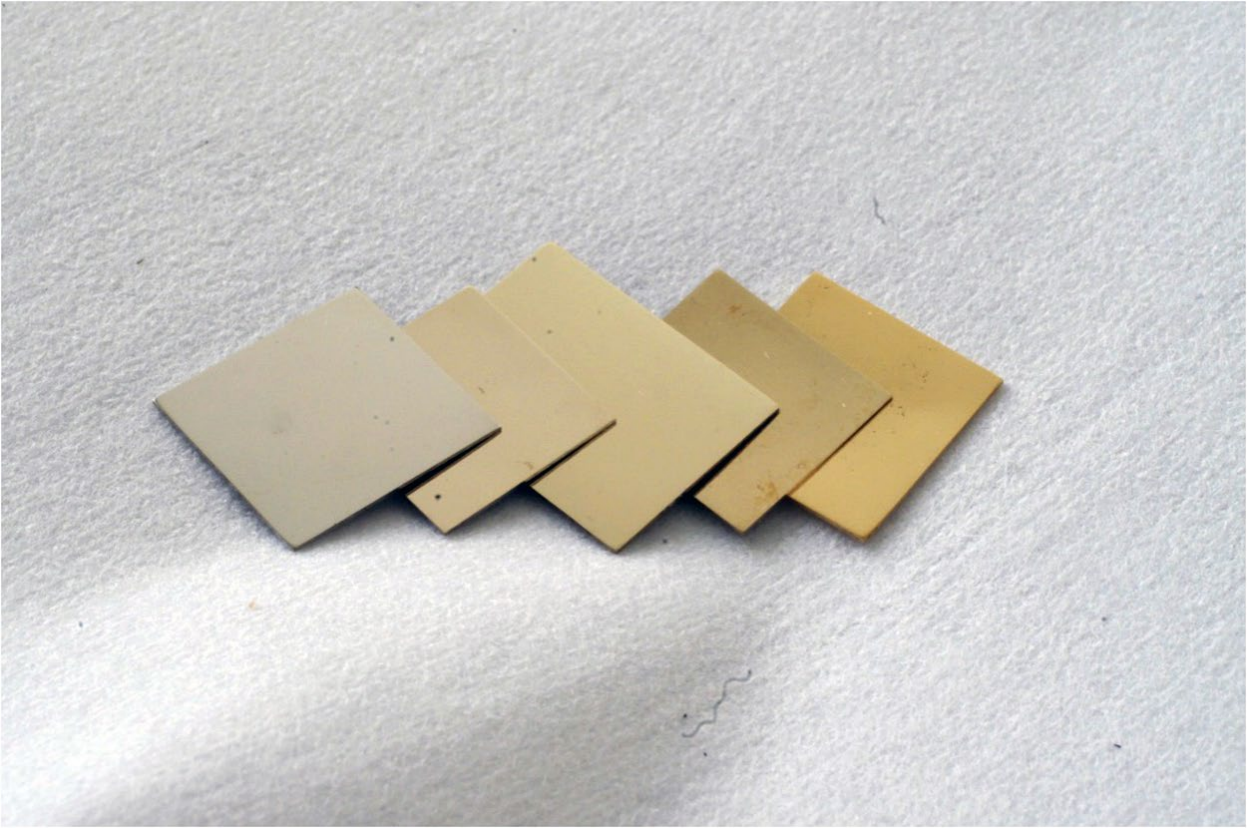
Plate coupons of the primary-Au, MGMC, and metallic glass alloys. From left to right, plate coupons of the monolithic metallic glass Au_50_Cu_25.5_Ag_3_Pd_3_Si_18.5_, MGMCs Au_55.5_Cu_24.4_Ag_6.2_Pd_2_Si_11.9_, Au_58_Cu_24_Ag_7.5_Pd_1.5_Si_9_ and Au_60_Cu_23.5_Ag_9.1_Pd_1_Si_6.4_, and primary-Au alloy Au_65.2_Cu_22.4_Ag_12.4_. The coupons reveal an alloy chromaticity that is compositionally-varying (from pale-gold to yellow-gold) yet visually unresolved to the naked eye.

Finely-polished 20 mm × 20 mm × 1 mm plate coupons of MGMCs Au_60_Cu_23.5_Ag_9_Pd_1.1_Si_6.4_, Au_58_Cu_24_Ag_7.5_Pd_1.5_Si_9_, and Au_55.5_Cu_24.4_Ag_6.2_Pd_2_Si_11.9_, monolithic metallic glass Au_50_Cu_25.5_Ag_3_Pd_3_Si_18.5_, and primary-Au Au_65.2_Cu_22.4_Ag_12.4_ were prepared. The coupons are displayed in Fig. 4. Visually, the MGMC coupons appear macroscopically homogeneous, while their surface color appears to be visually uniform and intermediate between those of the crystalline and the metallic glass coupons. The coupon surface color from left to right appears to transition from the pale-gold color of the monolithic metallic glass to the yellow-gold color of the primary-Au alloy. That is, the MGMCs appear to display an increasingly yellower color as *x* decreases from 1 to 0, reflecting the corresponding decrease in the molar (or volume) fraction of metallic glass phase from 100% to 0.

The CIELAB color coordinates of the 20 mm × 20 mm × 1 mm plate coupons are measured in color space by an optical spectrophotometer on the plate coupons (see Methods). The measured CIELAB coordinates are listed in Table S2 (see Supplemental Information). The CIELAB coordinates of the primary-Au Au_65.2_Cu_22.4_Ag_12.4_ are consistent with a yellow color, and falls within the “yellow” region of the Au-Cu-Ag color map (see Fig. S1, Supplemental Information). This demonstrates that the color of the primary-Au phase in the MGMCs is fixed by the choice of the Ag and Cu concentrations to a custom yellow color. In principle, by choosing different Cu and Ag concentrations one may potentially achieve any color in the Au-Cu-Ag color map. On the other hand, the CIELAB coordinates of the monolithic metallic glass Au_50_Cu_25.5_Ag_3_Pd_3_Si_18.5_ are consistent with a pale white color. The pale white color is mostly a consequence of a high Si content along with modest Pd content, as both Si and Pd are known to “bleach” the color of gold alloys. Changing the concentrations of Ag and Cu in the overall alloy in order to influence the color of the primary-Au phase, as discussed above, has little impact on the color of the metallic glass phase, which likely remains pale white due to the high Si and Pd contents.

**Figure 5:**
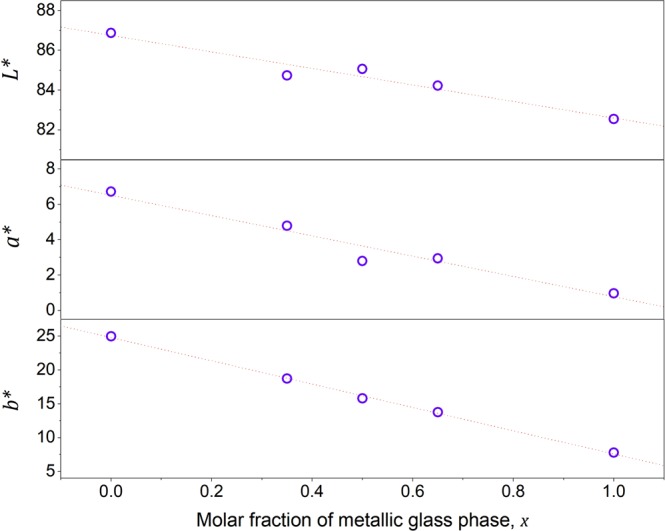
Color Coordinates vs. Metallic Glass Molar Fraction. Plots of CIELAB L*, a*, and b* coordinates vs. the metallic glass molar fraction *x*. Roughly linear dependencies are revealed, suggesting that the overall color of the composite follows the rule of mixtures, which implies that the microstructures of the composites are visually unresolved.

The CIELAB coordinate L*, which quantifies the “luminosity” or “reflectivity” of the alloy, decreases slightly with increasing *x* from 87.43 characterizing the primary-Au (*x* = 0) to 82.55 corresponding to the metallic glass (*x* = 1.0). Likewise, CIELAB coordinate a*, which quantifies the “red-green” chromaticity of the alloy, decreases with increasing *x* roughly monotonically from 6.72 corresponding to the primary-Au (*x* = 0) to 0.97 characterizing the metallic glass (*x* = 1.0). But CIELAB coordinate b*, which quantifies the “blue-yellow” chromaticity of the alloy, appears to decrease rather broadly with increasing *x*, from 24.96 characterizing the primary-Au (*x* = 0) to 7.77 corresponding to the metallic glass (*x* = 1.0). This demonstrates that by varying *x* from 0 to 1, which amounts to varying the molar (or volume) fraction of the metallic glass phase in the composite from 0% to 100%, one may very effectively control the yellow chromaticity of the MGMC by controlling the CIELAB b* coordinate. Plots of CIELAB L*, a*, and b* coordinates vs. *x* are presented in Fig. 5. The roughly linear dependencies suggest that the overall color of the composite follows the rule of mixtures, which further implies that the microstructures of the composites are indeed visually unresolved.

Vickers hardness measurements were also performed on the 20 mm × 20 mm × 1 mm plate coupons (see Methods). Like the color coordinates, the hardness of the MGMCs is also found to vary with *x* in a manner consistent with the rule of mixtures. Specifically, the MGMCs with *x* = 0.35, 0.5, and 0.65 display Vickers hardness values of 220 HV, 250 HV, and 295 HV, which increase fairly linearly from the value of the primary-Au of 120 HV to that of the metallic glass of 350 HV (see Methods and Table S3, Supplemental Information). The hardness values of the as-quenched MGMCs represent 85%, 125%, and 145% improvements over that of the as-quenched primary Au_65.2_Cu_22.4_Ag_12.4_. These improvements far exceed those achievable by conventional heat treatment (e.g. age hardening) of primary-Au alloys, which typically do not exceed 50% (ref.^25^). Hence, the present MGMC concept of controlling color is also a very effective approach to promote hardness.

In conclusion, a concept is introduced for designing color in polychromatic metallic glass-forming alloy systems with large lattice mismatch. The concept relies on forming composites consisting of a metallic glass matrix and finely dispersed gold microdendrites (MGMCs), with the matrix and dendrites being of dissimilar color but the composite displaying an overall visually-unresolved average color. Here we exploit the large lattice mismatch and broad chromaticity range in the Au-Si metallic glass-forming system to design a series of Au-based MGMCs. The developed MGMCs display uniform overall yellow colors within a broad chromaticity range and high overall hardness. The approach may potentially be expanded to other polychromatic metal alloy systems, leading to a new generation of metals that combine advanced performance with cosmetic attributes.

## Methods

Alloy ingots are produced by inductive melting of the appropriate amounts of elemental constituents in a quartz tube under inert atmosphere. The purity levels of the constituent elements were as follows: Au 99.99%, Cu 99.995%, Ag 99.95%, Pd 99.95%, and Si 99.9999%. Rod samples and plate coupons are produced by melting the alloy ingots in a furnace at 950 °C under high purity argon inside quartz cylindrical or rectangular tubes having 0.5 mm thick walls, and rapidly quenching in a room-temperature water bath. Rod cross sections and plate surfaces were finely polished to 1 μm diamond mirror finish.

The amorphicity/crystallinity of the metallic glass and crystalline phases was investigated by x-ray diffraction analysis with Cu*K*α radiation, while phase transition thermodynamics of were investigated by differential scanning calorimetry at a scan rate of 20 K/min

CIELAB color coordinates were measured on 20 mm × 20 mm × 1 mm plate coupons using a Konica Minolta CM-700d spectrophotometer with aperture size of 8 mm. Reported values are averages between measurements performed at the four corners of each plate coupon.

Vickers hardness (HV0.5) was measured on 20 mm × 20 mm × 1 mm plate coupons using a Vickers microhardness tester with an indenter having a width of 40 μm. Since the average size of the microstructural features in the MGMCs is below 5 μm, the indenter size is considered sufficiently large to provide a representative average hardness. Eight tests were performed where micro-indentions were inserted on a flat and polished cross section of a rod. A load of 500 g and a duel time of 10 s were used.

## Supplementary information

Supplementary Information

## Data Availability

All data generated or analyzed during this study are included in this published article (and its Supplementary Information files).

## References

[1] Ashby MF, Greer AL (2006). Metallic Glasses as Structural Materials. Scripta Mater..

[2] Demetriou MD (2011). A Damage Tolerant Glass. Nat. Mater..

[3] Guo SF (2013). Novel Centimeter Sized Fe-Based Bulk Metallic Glass with High Corrosion Resistance in Simulated Acid Rain and Sea Water. J. Non-Cryst. Sol..

[4] Kumar G, Tang HX, Schroers J (2009). Nanomolding with Amorphous Metals. Nature.

[5] Schroers J (2010). Processing of Bulk Metallic Glasses. Adv. Mater..

[6] Schroers J (2011). Thermoplastic Blowmolding of Metals. Materials Today.

[7] Johnson WL (2011). Beating Crystallization in Glass-Forming Metals by Millisecond and Processing. Science.

[8] Kaltenboeck G (2014). Accessing Thermoplastic Processing Windows in Metallic Glasses using Rapid Capacitive Discharge. Scientific Reports.

[9] Kaltenboeck G, Demetriou MD, Roberts S, Johnson WL (2016). Shaping Metallic Glasses by Electromagnetic Pulsing. Nat. Commun..

[10] Na JH (2014). Compositional Landscape for Glass Formation in Metal Alloys. PNAS.

[11] Johnson WL, Na JH, Demetriou MD (2016). Quantifying the Origin of Metallic Glass Formation. Nat. Commun..

[12] Ritchie RO (2011). The Conflicts Between Strength and Toughness. Nat. Mater..

[13] Hays CC, Kim CP, Johnson WL (2000). Microstructure Controlled Shear Band Pattern Formation and Enhanced Plasticity of Bulk Metallic Glasses Containing *In Situ* Formed Ductile Phase Dendrite Dispersions. Physical Review Letters.

[14] Hofmann DC (2008). Designing Metallic Glass Matrix Composites with High Toughness and Tensile Ductility. Nature.

[15] Launey ME (2009). Fracture Toughness and Crack-Resistance Curve Behavior in Metallic Glass Matrix Composites. Applied Physics Letters.

[16] Schroers J, Johnson WL (2004). Highly Processable Bulk Metallic Glass-Forming Alloys in the Pt-Co-Cu-Ni-P System. Applied Physics Letters.

[17] Schroers J, Lohwongwatana B, Johnson WL, Peker A (2005). Gold Based Bulk Metallic Glass. Applied Physics Letters.

[18] Schroers J, Lohwongwatana B, Johnson WL, Peker A (2007). Precious Bulk Metallic Glasses for Jewelry Applications. Materials Science & Engineering A.

[19] Zhang W (2009). New Au-Based Bulk Glassy Alloys with Ultralow Glass Transition Temperature. Scripta Mater..

[20] Guo H (2009). Glass-Forming Ability and Properties of New Au-Based Glassy Alloys with Low Au Concentrations. Materials Transactions.

[21] Mozgovoy S, Heinrich J, Klotz UE, Busch R (2010). Investigation of Mechanical, Corrosion, and Optical Properties of an 18 Carat Au-Cu-Si-Ag-Pd Metallic Glass. Intermetallics.

[22] Eisenbart M, Klotz UE, Busch R, Gallino I (2014). On the Abnormal Room Temperature Tarnishing of an 18 Carat Gold Bulk Metallic Glass Alloy. Journal of Alloys and Compounds.

[23] Lee SY (2007). Pseudo-Binary Phase Diagram for Zr-Based *In Situ* β-Phase Composites. Journal of Materials Research.

[24] Lee ML, Li Y, Schuh CA (2004). Effect of a Controlled Volume Fraction of Dendritic Phases on the Tensile and Compressive Ductility in La-based Metallic Glass Matrix Composites. Acta Mater..

[25] McDonald AS, Sistare GH (1978). The Metallurgy of Some Carat Gold Jewellery Alloys. Gold Bulletin.

