# Supplementary material for: Designing color in metallic glass
Source: Sci Rep. 2019 Mar 1;9:3269. doi: 10.1038/s41598-019-40014-w (PMC6397249; doi:10.1038/s41598-019-40014-w)
Supplement: Supplementary file 1 — Supplementary Information [file 41598_2019_40014_MOESM1_ESM.pdf]

SUPPLEMENTARY INFORMATION

Designing color in metallic glass

Jong Hyun Na[1], Kyung Hee Han[1], Glenn R. Garrett[1], Maximilien E. Launey[1], Marios D. Demetriou[1,2,a], and William L. Johnson[1,2]

[1] Glassimetal Technology, Inc., Pasadena, CA 91107

[2] Department of Applied Physics and Materials Science, California Institute of Technology, Pasadena, CA 91125

[a] Author to whom correspondence should be addressed: marios@glassimetal.com

Table S1. Glass transition temperature T_g, crystallization temperature T_x, solidus temperature T_s, and liquidus temperature T_l for the primary-Au, MGMCs and metallic glass alloy having compositions according to Eq. (1) corresponding to *x* values of 0, 0.35, 0.5, 0.65, and 1.

**Composition (at.%)**	***x***	T_g (°C)	T_x (°C)	T_s (°C)	T_l (°C)
$Au_{65.2}Cu_{22.4}Ag_{12.4}$	0	N/A	N/A	917.8	946.4
$Au_{60}Cu_{23.5}Ag_{9}Pd_{1.1}Si_{6.4}$	0.35	118.4	160.4	350.6	857.8
$Au_{58}Cu_{24}Ag_{7.5}Pd_{1.5}Si_{9}$	0.50	115.1	159.1	348.6	800.1
$Au_{55.5}Cu_{24.4}Ag_{6.2}Pd_{2}Si_{11.9}$	0.65	116.8	161.1	347.2	718.6
$Au_{50}Cu_{25.5}Ag_{3}Pd_{3}Si_{18.5}$	1.0	112.6	168.7	344.4	376.9

Table S2. CIELAB color coordinates of the primary-Au, MGMCs, and metallic glass alloy having compositions according to Eq. (1) corresponding to *x* values of 0, 0.35, 0.5, 0.65, and 1.

**Composition (at.%)**	***x***	***L******	***a******	***b******
$Au_{65.2}Cu_{22.4}Ag_{12.4}$	0	86.87	6.72	24.96
$Au_{60}Cu_{23.5}Ag_{9}Pd_{1.1}Si_{6.4}$	0.35	84.73	4.79	18.71
$Au_{58}Cu_{24}Ag_{7.5}Pd_{1.5}Si_{9}$	0.50	85.06	2.80	15.80
$Au_{55.5}Cu_{24.4}Ag_{6.2}Pd_{2}Si_{11.9}$	0.65	84.22	2.94	13.75
$Au_{50}Cu_{25.5}Ag_{3}Pd_{3}Si_{18.5}$	1	82.55	0.97	7.77

Table S3. Vickers hardness of the primary-Au, MGMCs, and metallic glass alloy having compositions according to Eq. (1) corresponding to *x* values of 0, 0.35, 0.5, 0.65, and 1.

Composition (at.%)	*x*	Hardness (HV)
$Au_{65.2}Cu_{22.4}Ag_{12.4}$	0	119.5 ± 12.3
$Au_{60}Cu_{23.5}Ag_{9}Pd_{1.1}Si_{6.4}$	0.35	219.5 ± 5.5
$Au_{58}Cu_{24}Ag_{7.5}Pd_{1.5}Si_{9}$	0.50	250.1 ± 3.2
$Au_{55.5}Cu_{24.4}Ag_{6.2}Pd_{2}Si_{11.9}$	0.65	296.3 ± 5.5
$Au_{50}Cu_{25.5}Ag_{3}Pd_{3}Si_{18.5}$	1	351.4 ± 2.7

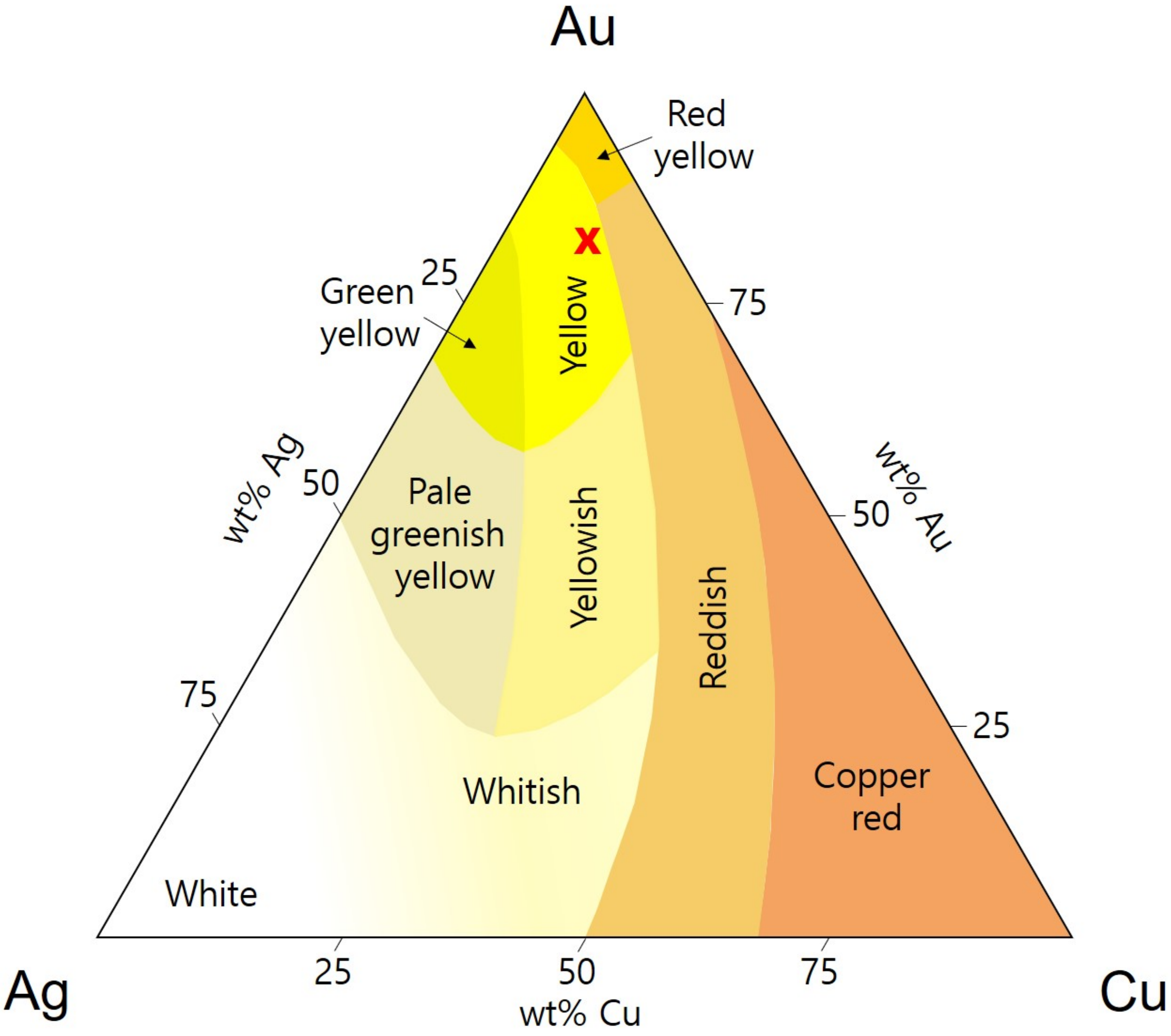

Figure S1 Color map of the Au-Ag-Cu system. Color-map of the ternary Au-Ag-Cu alloys system dividing the alloy composition space (in wt.%) into regions according to the optical appearance of the alloys. The primary-Au phase corresponding to x = 0 in Eq. (1), whose composition (in wt.%) is $Au_{82.3}Cu_{9.1}Ag_{8.6}$, is represented by the red cross superimposed on color map and appears to lie within the yellow color region.

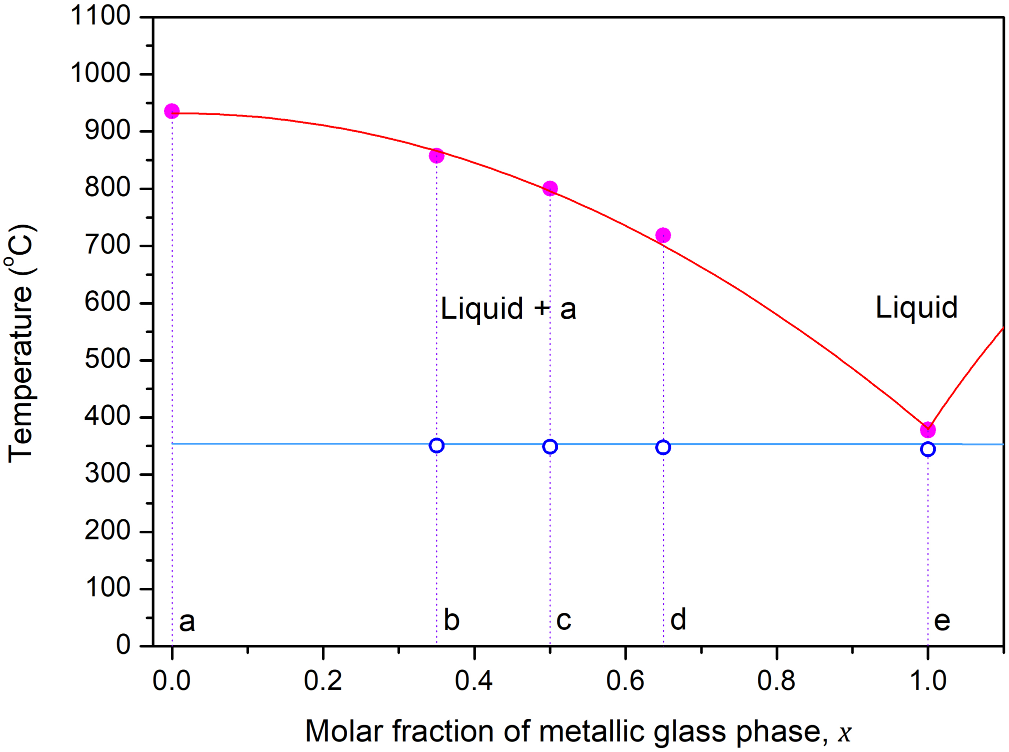

Figure S2 Pseudo-binary eutectic phase diagram. Pseudo-binary eutectic phase diagram. The phase diagram represents phase equilibria between primary $Au_{65.2}Cu_{22.4}Ag_{12.4}$ with x = 0 **(a)** and eutectic $Au_{50}Cu_{25.5}Ag_3Pd_3Si_{18.5}$ with x = 1 **(e)** in accord with the "tie line" construction described in Eq. (1). The compositions of MGMCs $Au_{60}Cu_{23.5}Ag_{9.1}Pd_1Si_{6.4}$ **(b)**, $Au_{58}Cu_{24}Ag_{7.5}Pd_{1.5}Si_9$ **(c)**, and $Au_{55.5}Cu_{24.4}Ag_{6.2}Pd_2Si_{11.9}$ **(d)** corresponding to x values of 0.35, 0.5, and 0.65 in Eq. (1) are also designated. The data for the eutectic temperature of 350°C along with the data for the receding liquidus temperature as a function of x are plotted. The liquidus temperature data are fitted using a 2nd order polynomial.
